# *Olea europaea* Suppresses Inflammation by Targeting TAK1-Mediated MAP Kinase Activation

**DOI:** 10.3390/molecules26061540

**Published:** 2021-03-11

**Authors:** Chaoran Song, Mi-Yeon Kim, Jae Youl Cho

**Affiliations:** 1 Department of Integrative Biotechnology, and Biomedical Institute for Convergence at SKKU (BICS), Sungkyunkwan University, Suwon 16419, Korea; songchaoran115@163.com; 2 School of Systems Biomedical Science, Soongsil University, Seoul 06978, Korea

**Keywords:** *Olea europaea*, anti-inflammatory effect, TAK1, MAP kinases

## Abstract

Possessing a variety of medicinal functions, *Olea europaea* L. is widely cultivated across the world. However, the anti-inflammatory mechanism of *Olea europaea* is not yet fully elucidated. In this study, how the methanol extract of the leaves of *Olea europaea* (Oe-ME) can suppress in vitro inflammatory responses was examined in terms of the identification of the target protein. RAW264.7 and HEK293T cells were used to study macrophage-mediated inflammatory responses and to validate the target protein using PCR, immunoblotting, nuclear fraction, overexpression, and cellular thermal shift assay (CETSA) under fixed conditions. Oe-ME treatment inhibited the mRNA expression levels of cyclooxygenase (COX)-2, matrix metallopeptidase (MMP)-9, and intercellular adhesion molecule-1 (ICAM-1) in activated RAW264.7 cells. Oe-ME diminished the activation of activator protein (AP)-1 and the phosphorylation of its upstream signaling cascades, including extracellular signal regulated kinase (ERK), mitogen-activated protein kinase kinase 1/2 (MEK1/2), c-Jun N-terminal kinase (JNK), mitogen-activated protein kinase kinase 3/6 (MKK3/6), p38, MKK7, and transforming growth factor-β-activated kinase 1 (TAK1), in stimulated-RAW264.7 cells. Overexpression and CETSA were carried out to verify that TAK1 is the target of Oe-ME. Our results suggest that the anti-inflammatory effect of Oe-ME could be attributed to its control of posttranslational modification and transcription of TAK1.

## 1. Introduction

Inflammation is a defense response of living cells to inflammatory factors, local damage, bacteria, virus, and fungi. Acute inflammation usually involves changes such as redness, swelling, and heat and is often accompanied by systemic reactions such as fever and leukocytosis [1,2]. Toll-like receptors (TLRs) of innate immune cells recognize extracellular stimuli and produce inflammatory responses. Lipopolysaccharides (LPS), pam3Cys-Ser-(Lys)4 (Pam3CSK4), and polyinosinic:polycytidylic acid (poly (I:C)) are TLR agonists that induce specific activation of TLR4, TLR2, and TLR3, respectively [3,4].

The recognition between TLRs and their ligands stimulates the recruitment of intracellular adaptors such as MyD88 and TRIF, which subsequently provoke interleukin 1 receptor associated kinase 1 (IRAK1), a member of the IRAK family. IRAK1 then mediates formation of TRAF6-TAK1-TAB2 complex, which is a vital procedure to activate transforming growth factor-β-activated kinase 1 (TAK1) [5,6]. Once TAK1 is activated, a sequential signaling cascade composed of mitogen-activated protein kinase kinases (MAPKKs) and kinase IKK is activated. Then, MAPKKs or IKK phosphorylate MAPK [c-Jun-N-terminal kinase (JNK), extracellular signal-regulated kinase1/2 (ERK1/2), and p38] or the inhibitor of κBα (IκBα) to activate activator (AP)-1 or NF-κB for transcription of pro-inflammatory genes [7,8]. The members of MAPKKs include MEK1/2, MEK5, MKK4/7, and MKK3/6, which phosphorylate and activate subsequent downstream enzyme MAPKs. JNK, ERK1/2/5, and p38 subsequently phosphorylate AP-1 subunits (e.g., c-Fos and c-Jun) to induce translocation of activator protein (AP-1) by the interaction between subgroups of MAPKs and the amino-terminal activation domain of c-Fos and c-Jun [7,9].

Many critical proinflammatory genes, such as inducible nitric oxide synthase (iNOS), cyclooxygenase (COX)-2, adhesion molecules [intercellular adhesion molecule-1 (ICAM-1)], chemokines [e.g., monocyte chemotactic protein-1 (MCP-1)], and cytokines [tumor necrosis factor-α (TNF-α) and interleukins], have been transcribed [9]. Nitric oxide (NO), prostaglandin E2 (PGE2), cytokines, chemokines, and adhesion molecules play crucial roles in the regulation of macrophage-mediated inflammatory reaction [10,11]. Although the inflammatory response is vital to body homeostasis, excessive and sustained responses contribute to cancer, rheumatoid arthritis, diabetes, and arteriosclerosis [1].

*Olea europaea L.* is a plant that is commonly used as ethnopharmacological food and medicine in the Mediterranean, North America, and Australia. *Olea europaea* L. is traditionally used to treat hypertension, inflammatory diseases, cardiovascular diseases, cancer, and obesity [12]. Due to its various biophenols and other bioactive ingredients, it has been identified as a functional food. Special attention is paid to the biological and pharmacological properties of those compounds. Oleuropein, secoiridoids, hydroxytyrosol, caffeic acid, *p*-coumaric acid, vanillic acid, and iridoids from *Olea europaea* L. have been isolated and demonstrated to possess variety of pharmacological activities such as antioxidant [13], anti-inflammatory, antimicrobial, anti-atherosclerosis [14], and antiviral properties [15]. Our previous results suggest that the methanol extract of *Olea europaea* L. (Oe-ME) and its components, kaempferol (KFL), quercetin (QCN), and luteolin, reduce the activation of Src and Syk in the NF-κB signaling pathway and protects cells from excessive inflammation. However, the molecular mechanisms regarding its anti-inflammatory effect in other signaling pathways remains unknown. In this study, we aimed to explore how Oe-ME exerts its anti-inflammatory effect with respect to the AP-1 signaling pathway.

## 2. Results

### 2.1. Cytotoxicity and the Effect of Oe-ME on Production of Inflammatory Genes

**Figure 1:**
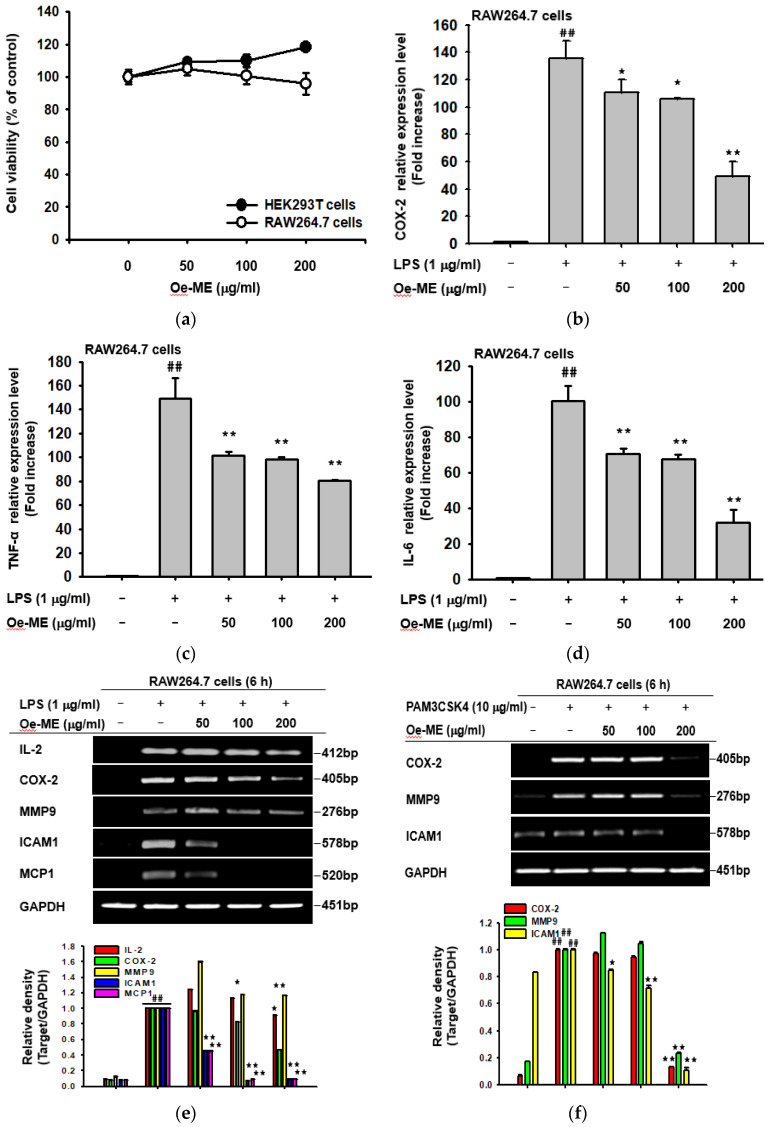

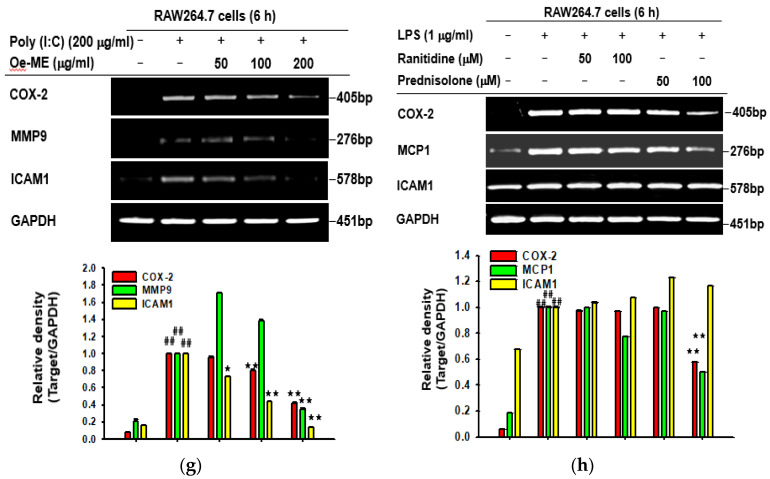
Cell viability and the effect of Oe-ME on mRNA expression of inflammation-related genes. (**a**) RAW264.7 and HEK293T cells were treated with indicated doses of Oe-ME (50, 100, and 200 μg/mL) and then incubated for 24 h. Cell viability was measured using an MTT assay. (**b**,**c**,**d**) After pretreatment with Oe-ME for 30 min, RAW264.7 cells were exposed to LPS and real-time PCR were employed to assess the production of TNF-α, COX-2, and IL-6. (**e**,**f**,**g**,**h**) RAW264.7 cells were incubated with LPS, poly (I:C), or pam3CSK4 in the absence or presence of various concentrations of Oe-ME. The mRNA expression levels of ICAM-1, IL-2, COX-2, MMP-9, and MCP1 were measured by RT-PCR. Prednisolone and ranitidine were used as positive and negative control drugs, respectively. Band intensity was measured and quantified using ImageJ. ## *p* < 0.01 compared with the normal group, and * *p* < 0.05 and ** *p* < 0.01 compared with the LPS, pam3CSK4, or poly (I:C) alone groups treated with the corresponding concentration of the vehicle (DMSO).

To address our experimental aims, we used two cell lines (RAW264.7 and HEK293T cells) in this study. RAW264.7 cells, mouse mononuclear macrophage-like cells, are immune cells that can show inflammatory responses to pathogens [16]. Since RAW264.7 cells show low transfection efficiency, we employed HEK293T cells displaying higher transfection efficiency to validate putative target protein by overexpression of its gene [17]. We found that Oe-ME had no cytotoxicity on RAW264.7 and HEK293T cells at the tested concentrations (Figure 1a). LPS enables macrophages to release important inflammatory cytokines and mediators, which is vital to activate subsequent immune responses. Therefore, in our study, LPS was utilized to stimulate RAW267.4 cells. The role of Oe-ME in the production of inflammatory genes was explored by RT-PCR. The mRNA expression levels of IL-2, IL-6, COX-2, MMP-9, ICAM-1, TNF-α, and MCP1 were sharply elevated by LPS, while their expression was decreased by Oe-ME treatment in a dose-dependent manner (Figure 1e). The same pattern was obtained of the expression levels of COX-2, TNF-α, and IL-6 through quantitative real-time PCR (Figure 1b–d). The mRNA production of COX-2, TNF-α, and IL-6 was significantly (*p* < 0.05 and 0.01) decreased by Oe-ME. Pam3CSK4 and poly (I:C) were also applied to induce inflammation. After stimulation, the enhanced expression of COX-2, MMP-9, and ICAM1 was significantly (*p* < 0.05 and 0.01) diminished by Oe-ME treatment (Figure 1f,g). Meanwhile, whether expression levels of COX-2, MCP1, and ICAM1 can be modulated by prednisolone (a steroidal anti-inflammatory drug: positive control drug) or ranitidine (an H2 receptor blocker: negative control drug) was examined. As Figure 1h shows, 100 μM of prednisolone but not ranitidine significantly (*p* < 0.01) reduced the expression of both COX-2 and MCP1 but not ICAM1.

### 2.2. Roles of Oe-ME in Transcriptional Activation of AP-1

**Figure 2:**
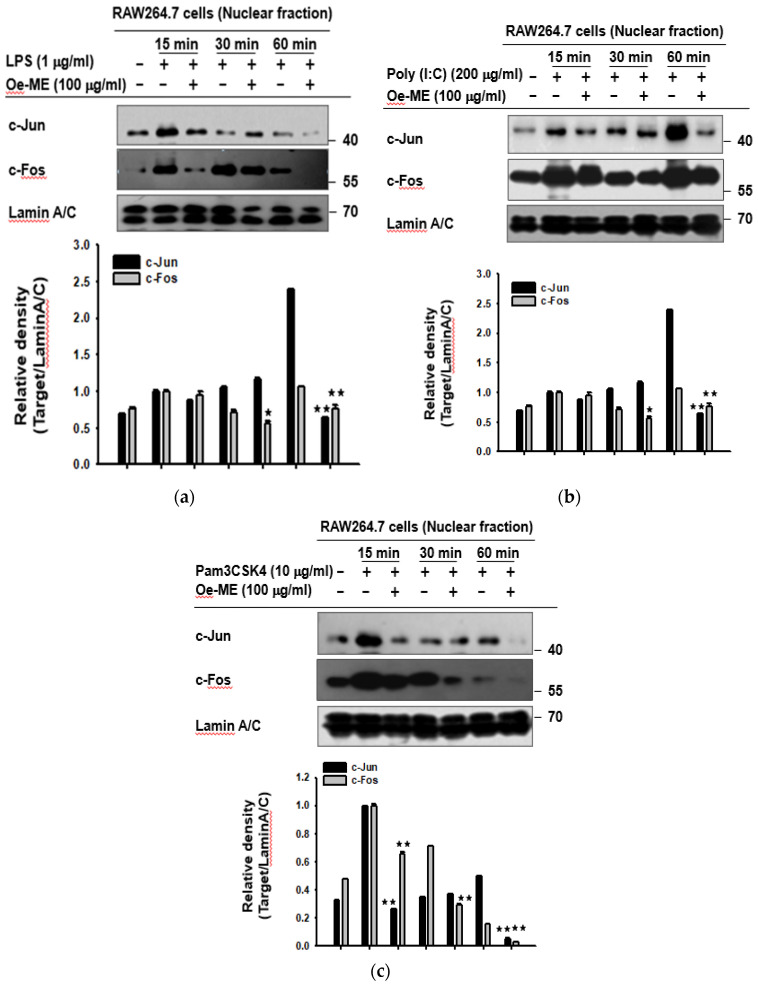
The role of Oe-ME in the transcriptional activation of AP-1. (**a**) After being pre-incubated with Oe-ME for 30 min, RAW264.7 cells were stimulated by LPS for intended periods of time (15, 30, and 60 min). Levels of c-Fos, c-Jun, and Lamin A/C in nuclear fractions were determined by immunoblotting analysis. (**b**,**c**) Similarly, TLR agonists of TLR2 and -3 were used to induce RAW264.7 cells. c-Fos, c-Jun, and Lamin A/C from nuclear fractions were subjected to immunoblotting analysis. Band intensity was measured and quantified using ImageJ. * *p* < 0.05 and ** *p* < 0.01 compared with LPS alone treated with the corresponding concentration of the vehicle (DMSO).

It has been shown that c-Fos is capable of interacting with c-Jun, which is essential for inducing transcription of AP-1 responsive genes [18]. RAW264.7 cells were collected after being activated by poly (I: C), pam3CSK4, or LPS. The nuclear fractions were extracted, and the protein levels of c-Fos and c-Jun were detected by immunoblotting. As expected, the protein expression levels of c-Fos and c-Jun were dramatically enhanced by these three inducers at all detected time points. Upon these activated situations, the elevated expression levels of c-Fos and c-Jun were significantly (*p* < 0.01) diminished by Oe-ME at 15, 30, and 60 min, while protein level of Lamin A/C, a nuclear envelope marker, was not significantly altered (Figure 2).

### 2.3. Role of Oe-ME in AP-1 Signal Transduction

**Figure 3:**
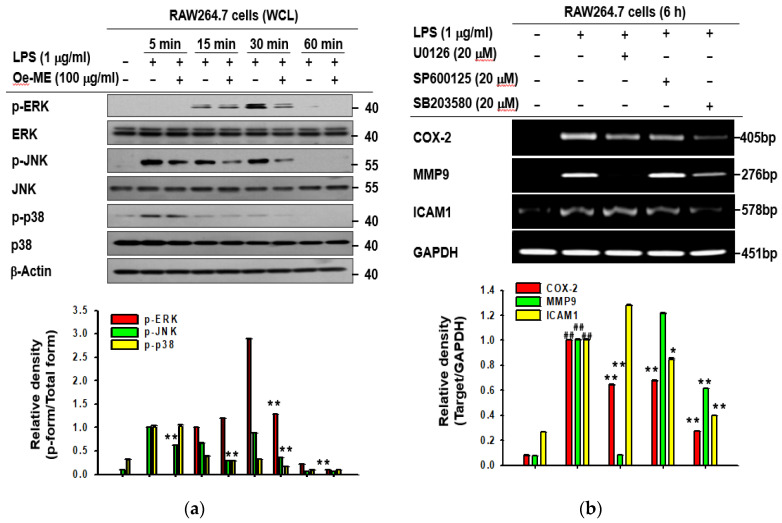

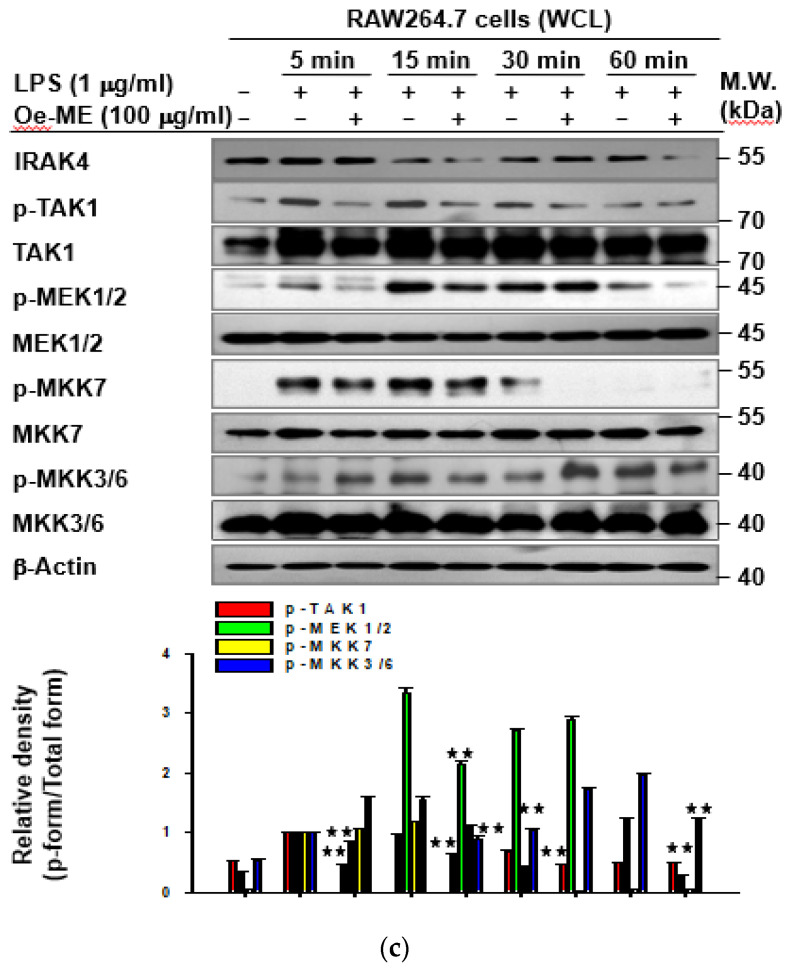
The effect of Oe-ME on mitogen activated protein kinase (MAPK) activation and its upstream signaling. (**a**) RAW264.7 cells were treated with Oe-ME or vehicle and were then induced by LPS for 5, 15, 30, and 60 min. The phosphorylated and total forms of MAPKs and MAPKKs in the whole cell lysates were detected. (**b**) MAPK inhibitors were used to verify the role of MAPKs on LPS stimulation through RT-PCR. (**c**) RAW264.7 cells were treated with Oe-ME or LPS for indicated periods of time. Phospho- and total forms of MEK1/2, MKK7, and MKK3/6 were determined by immunoblotting analysis. Band intensity was measured and quantified using ImageJ. ## *p* < 0.01 compared with the normal group, and * *p* < 0.05 and ** *p* < 0.01 compared with LPS alone treated with the corresponding concentration of the vehicle (DMSO).

To explore which molecule can be targeted by Oe-ME in the AP-1 signaling pathway, RAW264.7 cells were exposed to LPS, and the phosphorylated and total levels of JNK, p38, and ERK were tested. A significant decrease (*p* < 0.01) of phosphorylated JNK was observed at 5, 15, 30, and 60 min. The phosphorylated level of ERK was significantly (*p* < 0.01) downregulated at 15 and 30 min, while the reduced level of p38 was detected at 5 and 30 min (Figure 3a). Next, we wanted to further demonstrate that JNK, p38, and ERK are essentially involved in AP-1-mediated gene production in LPS-induced RAW264.7 cells. The expression levels of MMP-9, COX-2, and ICAM1 were assessed after activated RAW264.7 cells were treated in the absence or presence of MAPK inhibitors (U0126, inhibitor of ERK; SP600125, inhibitor of JNK; SB203580, inhibitor of p38, respectively). The production of these inflammatory genes was blocked by MAPK inhibitors to varying degrees (Figure 3b). U0126 treatment significantly suppressed the levels of COX-2 and MMP-9, whereas SP600125 inhibited expression of COX-2 and ICAM1. Additionally, the production of ICAM1, COX-2, and MMP-9 was blocked by SB203580. Afterwards, the upstream proteins of MAPKs were determined. Further immunoblotting aimed to elucidate which MAPK upstream molecules were targeted by Oe-ME. Oe-ME treatment reduced the phosphorylated levels of MEK1/2 and MKK7 at most tested time points, and a decrease of *p*-MKK3/6 was detected at 15 and 60 min (Figure 3c). A reduction of phosphorylated TAK1 was also observed at 5, 15, and 30 min, whereas the expression level of IRAK4, a protein that activates TAK1, did not exhibit significant alterations at early times. These findings suggest that TAK1 is a pharmacological target of Oe-ME, rather than its upstream enzymes.

### 2.4. Effect of Oe-ME on TAK1 Activation

**Figure 4:**
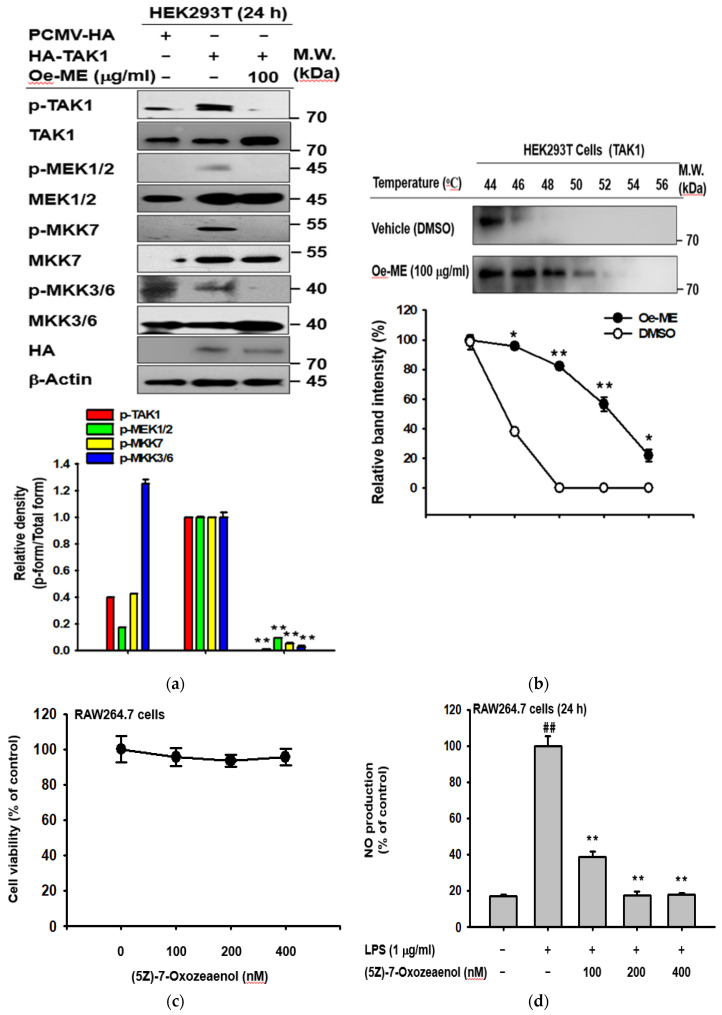

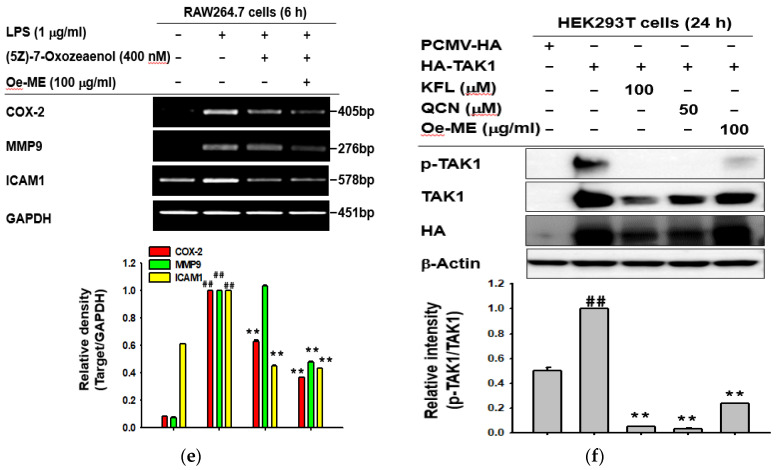
Confirmation of TAK1 as the target of Oe-ME. (**a**,**f**) HEK293T cells were transfected with either PCMV-HA or HA-TAK1 plasmids for 24 h and further treated with Oe-ME or its known ingredients, quercetin (QCN) and kaempferol (KFL). The target proteins, including the total TAK1, were detected by western blotting analysis. (**b**) After incubation with the HA-TAK1 plasmid, HEK293T cells were collected. Lysis from vehicle- and Oe-ME-treated cells were subjected to CETSA by western blotting analysis. (**c**) The viability of (5Z)-7-oxozeaenol-treated RAW264.7 cells for 24 h was determined by an MTT assay. (**d**) Upon LPS induction, the inhibitory effect of Oe-ME on NO production was explored by Griess assay. (**e**) The expression levels of COX-2, MMP-9, and ICAM1 in (5Z)-7-oxozeaenol-treated RAW264.7 cells during LPS exposure were measured by RT-PCR. Band intensity was measured and quantified using ImageJ. ## *p* < 0.01 compared with the normal group, and * *p* < 0.05 and ** *p* < 0.01 compared with LPS alone or the TAK1 overexpression group treated with the corresponding concentration of the vehicle (DMSO).

To assess the putative target (TAK1) of Oe-ME, an overexpression method was utilized to transfect HA-TAK1 plasmids to HEK2963T cells. As expected, Oe-ME also significantly (*p* < 0.01) reduced phosphorylation levels of TAK1, MEK1/2, MKK7, and MKK3/6, which were enhanced by LPS in the absence of Oe-ME (Figure 4a). The Cellular Thermal Shift Assay (CETSA) is a technology used to evaluate the interactions between target proteins and compounds based on inherent thermal stability in living cells. In this study, the intended temperatures are 44 °C, 46 °C, 48 °C, 50 °C, 52 °C, 54 °C, and 56 °C. Oe-ME treatment exhibited substantial shifts of the thermal stability of TAK1 (Figure 4b). Upon addition of Oe-ME, we clearly observed that TAK1 has better thermal stability, indicating the participation of the target. Meanwhile, to confirm whether TAK1 is a functionally important enzyme in LPS-mediated inflammatory responses, we employed the pharmacological inhibitor (5*Z)*-7-oxozeaenol known to form a covalent complex with TAK1 and irreversibly suppress the ATPase activity of TAK1 [19,20]. As shown in Figure 4c–e, LPS-induced upregulation of both inflammatory genes (COX-2, MMP-9, and ICAM1) and NO level was significantly (*p* < 0.01) abrogated by 5*Z*-7-oxozeaenol treatment, without obvious cytotoxicity. Interestingly, co-treatment of Oe-ME with (5*Z)*-7-oxozeaenol showed suppressed expression of COX-2, MMP9, and ICAM1 (Figure 4e). Finally, to determine which components identified from Oe-ME contribute to suppressing TAK1 activity of Oe-ME, we applied these compounds to TAK1-transfected HEK293T cells. As Figure 4f shows, KFL and QCN strongly reduced the level of p-TAK1 without altering the total TAK1 level.

## 3. Discussion

Numerous studies clarified the pharmacological function of *Olea europaea* because of its ethnopharmacological importance. Our group has also recently reported that the methanol extract of *Olea europaea* (Oe-ME) plays an anti-inflammatory role through the NF-κB signaling pathway [21]. In this paper, we aimed to further explore how Oe-ME attenuates inflammatory effects with respect to the AP-1 pathway.

Macrophages and dendritic cells secrete various cytokines and mediators, including interleukins, TNF-α, and chemokines. Chemokines are important in the aspect of mediating movement of mononuclear cells by activating G protein coupled receptors (GPCRs), engendering the adaptive immune response. Migrated cells are directed by chemokines to the sites of inflammation along the chemokine gradient. MCP-1, one of the key chemokines, regulates migration and infiltration of monocytes and macrophages [22]. Found in the membranes of leukocytes and endothelial cells, ICAM1 participates in cell adhesion, migration of leukocytes to sites of inflammation, and activation of lymphocytes [23]. MMP-9 acts broadly in inflammation to regulate barrier function and inflammatory cytokines through activation of its transcription, which is mediated by AP-1. [24]. The mRNA levels of the above related genes were tested to demonstrate that Oe-ME could mediate inflammatory responses in transcriptional regulation. Consistent with our assumption, Oe-ME treatment markedly decreased the production of inflammatory cytokines and mediators in a concentration-dependent manner according to both RT-PCR and quantitative real-time PCR, as shown in the case of prednisolone, a positive steroidal anti-inflammatory drug (Figure 1). A similar inhibitory trend was observed under different induction conditions by LPS, pam3CSK4, and poly (I:C), suggesting that Oe-ME can be applied for inflammatory responses generated by G(−)and G(+) bacteria.

Consistent with the above data, Oe-ME also decreased the nuclear protein levels of c-Fos and c-Jun under induction by different TLR ligands (Figure 2). Overall, these results suggest that upstream signal molecules which increase nuclear translocation of c-Jun/c-Fos, subunits of AP-1, could be the target of Oe-ME. Furthermore, immunoblotting was carried out to elucidate which molecules are involved in the Oe-ME-mediated anti-inflammatory effect in LPS-stimulated macrophages. Possessing AP-1 binding sites, three major MAPK pathways lead to AP-1 activation: ERK, p38, and JNK [25,26,27,28]. In our study, phosphorylated ERK, MEK1/2, MKK3/6, JNK, MKK7, and p38 were downregulated by Oe-ME treatment (Figure 3). Interestingly, the inhibitory effect on phosphorylated proteins of Oe-ME occurred at different stimulation time points—for instance, p-p38 at 5 min; p-JNK at 5, 15, and 30 min; and p-ERK at 30 min. This may indicate that various active ingredients in the extract play respective roles to target these enzymes separately. Indeed, KFL, identified in the methanol extract of *Olea europaea*, was reported to possess potent anti-inflammatory properties and functions as an inhibitor of inflammatory signaling composed of protein kinase C and MAPK [29,30]. QCN suppresses JNK/AP-1 signaling in a time-dependent manner in acetaminophen-induced liver damage [31]. Additionally, luteolin can effectively reduce proliferation of osteoarthritis cartilage cells, suppress the expression of JNK and p38 in cartilage cells, and inhibit the production of NO, TNF-α, and IL-6 [32,33]. Luteolin suppresses phosphorylation of ERK in the MAPK signaling pathway to induce apoptosis in the gastric cancer cell line BGC-823 [34]. Based on these reports [31,32,33,34], it is considerable that QCN, KFL, and luteolin could be major components in suppression of the MAPK pathway. In addition, we have also identified that oleuropein and hydroxytyrosol are included almost 50% and 4.3% of phenolic compounds in olive leaves [35]. Since oleuropein, but not hydroxytyrosol, was reported to suppress activation of ERK1/2 and JNK [36], there is a possibility that this compound could contribute to the inhibition of AP-1 pathway by Oe-ME. In future experiments, whether this compound can block the AP-1 pathway under our conditions will be further tested.

Inhibition of phosphorylated TAK1 was detected at 5, 15, and 30 min, whereas IRAK4 was not affected by Oe-ME treatment, suggesting that Oe-ME can modulate TAK1 activity via posttranslational modification (Figure 3c). In line with our expectations, this hypothesis is supported by the determination of p-MEK1/2, p-TAK1, p-MKK7, and p-MKK3/6 in HEK293T cells overexpressing the HA-TAK1 plasmid (Figure 4a). Consistent with this, usage of (5*Z)*-7-oxozeaenol confirmed that LPS-stimulated upregulation of COX-2, MMP-9, and ICAM1 expression and NO production was TAK1-dependent, without altering cell viability (Figure 4c–e). Finally, based on compound-induced thermal stability changes, the Cellular Thermal Shift Assay (CETSA) was applied to validate that Oe-ME binds to endogenous TAK1 in intact cells (Figure 4b). Our results clearly imply that Oe-ME targets TAK1 enzyme to modulate the AP-1 pathway, protecting cells from the effects of excessive inflammation. Moreover, as shown in Figure 4f, QCN, and KFL can reduce the phosphorylation of TAK1 raised under HA-TAK1 overexpression conditions, implying that these compounds are active components contributing to the anti-inflammatory activity of Oe-ME. In addition, inhibitory activities of QCN and KFL on TAK1 phosphorylation during LPS stimulation were also reported [37,38].

Pathophysiological roles of TAK1 have been demonstrated by a variety of animal and clinical studies. For example, neonatal hypoxic ischemic encephalopathy is an essential factor underlying neonatal death and disability. In the neonatal hypoxic ischemic encephalopathy models established in 10-day-neonatal Sprague Dawley rats, TAK1/NF-κB signaling was significant increased by oxidative stress and inflammatory responses [39]. TAK1 was found to be critically involved in sepsis-induced multiple organ dysfunction [40]. DSS-induced colitis was also revealed to be mediated by TAK1 activity in its pathology, containing clinical manifestations, histopathological damage, loss of tight junction function, and imbalanced intestinal microenvironment [41]. It also was reported that hepatocyte-specific deletion of TAK1 in Tak1 ΔHEP mice promotes liver fibrosis and hepatocellular carcinoma (HCC) [42]. These findings strongly support functional significance of TAK1 in many cellular and organic roles and imply that malfunction of this enzyme can produce health problems in human. Thus, it is expected that the TAK1-inhibitory potential of *Olea europaea* will be beneficial in treating various human diseases mediated by TAK1. Therefore, in terms of pathophysiological role of TAK1 in inflammatory responses, inhibition of TAK1 by *Olea europaea* might provide new safe and effective therapeutic approaches to prevent or cure various TAK1-mediated diseases.

**Figure 5:**
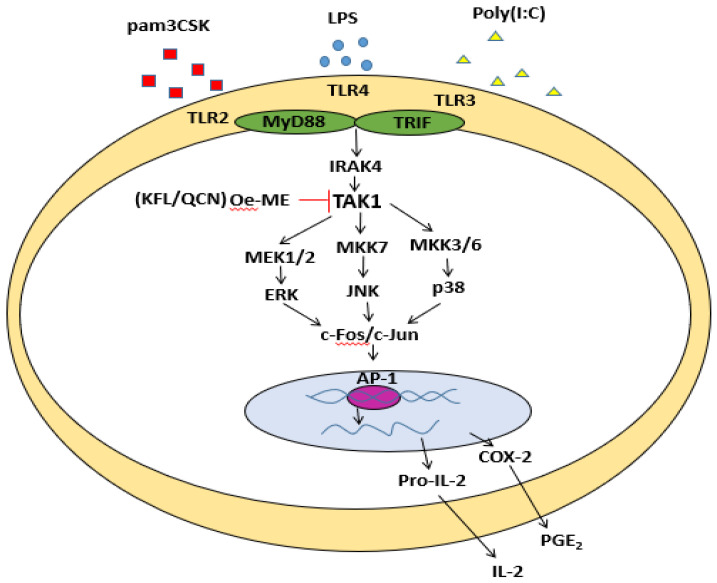
The schematic pathway of Oe-ME-regulated anti-inflammatory responses. Oe-ME inhibits TAK1 kinase activity in AP-1 pathway, leading to anti-inflammatory effects.

In summary, this study concluded that the methanol extract of *Olea europaea* (Oe-ME) attenuates the mRNA expression levels of IL-2, COX-2, MMP-9, ICAM1, and MCP1 in a concentration-dependent manner in RAW264.7 macrophages under TLR4, TLR3, and TLR2 activation conditions. Our results also indicated that Oe-ME is able to suppress TAK1 kinase activity, leading to reduced downstream signaling for activation of the AP-1 pathway, as summarized in Figure 5. Therefore, it is suggested that *Olea europaea* leaves can be further applied to develop anti-inflammatory remedy against TAK1/AP-1-mediated inflammatory diseases.

## 4. Materials and Methods

### 4.1. Materials and Reagents

Oe-ME was used as reported previously [21,35]. Briefly, it was prepared by extraction with 99.9% (*v*/*v*) methanol (yield: 17.4%) with individual components [KFL (content: 0.094%), QCN (0.038%), and luteolin (0.022%)] from leaves of the olive tree [21]. HPLC profile of this extract was already published previously [21]. Contents of other phenolic compounds [oleuropein isomer 1/2 (22.01 mg), secaloganoside isomer 1/2 (6.63 mg), quinic acid (4.04 mg), hydroxytyrosol (1.81 mg), and ligstroside (0.96 mg) in 1 g of leaves] were found in leaves of the olive tree [36]. Cell lines used in this study were obtained from ATCC (Rockville, MD, USA). Cell culture medium (DMEM and RPMI), phosphate-buffered saline (PBS), dimethyl sulfoxide (DMSO), fetal bovine serum (FBS), polyethylenimine (PEI), streptomycin, and penicillin were purchased from Gibco (Grand Island, NY, USA). Lipopolysaccharides (LPS), Poly (I:C), Pam3CSK4, prednisolone, ranitidine, and (5Z)-7-Oxozeaenol were obtained from Sigma-Aldrich (St. Louis, MO, USA). SB203580, U0126, and SP600125 were purchased from Calbiochem (La Jolla, CA, USA). Total and phospho-specific antibodies in this paper were obtained from Cell Signaling (Beverly, MA, USA). All the PCR primers were obtained from Macrogen (Seoul, Korea). As previously reported, plasmid constructs containing binding sites for TAK1 were purchased from Addgene (Cambridge, MA, USA) [43].

### 4.2. Oe-ME Preparation

Oe-ME was dissolved in 100% DMSO to make a 100 mg/mL stock solution and further diluted with DMSO to prepare 50 and 25 mg/mL doses. Finally, culture medium was used to prepare working solutions of 50, 100, and 200 μg/mL. The final concentration of DMSO in each dose was 0.2%. Normal and control groups [LPS, pam3CSK, or poly(I:C) alone] were treated with the same amount of vehicle (DMSO).

### 4.3. Cell Culture

RAW264.7 cells were cultured in RPMI medium containing 10% fetal bovine serum, whereas HEK293T cells were cultured in DMEM medium with 5% fetal bovine serum. Both media contained 1% antibiotics. RAW264.7 and HEK293T cells were maintained at 37 °C and 5% CO_2_. Subculture was performed every two or three days when the cells reached 70% confluence. The cells were collected with cell scrapers or trypsin for in vitro experiments.

### 4.4. Cytotoxicity Evaluation

HEK293T and RAW264.7 cells (1 × 10^6^ cells/mL) were seeded into 96-well plates and incubated for 18 h. The cell viability of Oe-ME on HEK293T and RAW264.7 cells as well as the cytotoxicity effect of (5Z)-7-Oxozeaenol on RAW264.7 cells were investigated by MTT assay [44,45].

### 4.5. Nitric Oxide (NO) Production

After pre-incubation (1 × 10^6^ cells/mL) for 18 h, RAW264.7 cells were treated with (5Z)-7-oxozeaenol (0–400 nM) for 30 min and then further stimulated with LPS (1 μg/mL) for 24 h. The NO level was determined by Griess assay [46].

### 4.6. Semi-Quantitative RT-PCR and Quantitative Real-Time PCR

RAW264.7 cells were planted into 6-well plates at A density of 1 × 10^6^ cells/mL. After exposure to Oe-ME for 30 min, RAW264.7 cells were further incubated with LPS for 6 h. The mRNA expression levels of genes related to inflammation (IL-2, IL-6, COX-2, TNF-α, MMP-9, MCP1, and ICAM-1) were determined. Prednisolone and ranitidine were used as positive and negative control drugs, respectively. To explore the role of MAPKs under LPS treatment, LPS was utilized to induce RAW264.7 cells in the absence or presence of MAPK inhibitors (U0126, SP600125, and SB203580). To elucidate the role of TAK1, LPS or (5Z)-7-oxozeaenol (400 nM) was used to treat RAW264.7 cells. The mRNA was isolated using TRI reagent^®^, and the total RNA was immediately subjected to cDNA synthesis. Then, PCR was conducted based on previous reports [47,48]. All sequences of primers in this paper are provided in Table 1.

### 4.7. Plasmid Transfection

Transfection was performed when HEK293T cells (2.5 × 10^5^ cells/mL) were grown to 70% confluence. A group of cells was transfected with pCMV-HA, while two groups were treated with HA-TAK1 plasmid using PEI (24 h). Then, the vehicle (0.2% DMSO) and Oe-ME (100 μg/mL) or individual flavonoids (QCN and KFL) were used to treat cells for another 24 h, and protein was extracted as previously described [49].

### 4.8. Preparation of Nuclear and Total Samples for Immunoblotting

RAW264.7 cells (5 × 10^6^ cells/mL) were pre-treated with Oe-ME for 30 min and then stimulated by LPS for a specific period. The cells were then collected, washed with cold PBS, and lysed in whole cell lysis buffer. Next, the pellet was obtained by centrifuging the lysates. As in a previous report, nuclear fractions were isolated [50]. The proteins in total and nuclear lysates were separated by SDS-PAGE and subjected to PVDF membranes. The membranes were blocked in 3% BSA for 1 h at room temperature, incubated with the specific primary antibody overnight at 4 °C, and subsequently incubated with a secondary antibody for another 1 h at room temperature. Phosphorylated and total forms of β-actin, c-Fos, JNK, Lamin A/C, p38, ERK, MKK4/7, c-Jun, MKK3/6, MEK1/2, TAK1, IRAK4, and HA were detected by the ECL system [51].

### 4.9. Cellular Thermal Shift Assay (CETSA)

HEK293T cells (2 × 10^6^ cells/mL) transfected with HA-TAK1 plasmid for 24 h were divided into two groups and treated with Oe-ME (100 μg/mL) or vehicle (0.2% of DMSO) for 1 h, respectively. The cells were then washed with cold PBS, pelleted, and resuspended in PBS with protease inhibitors. The resuspended cells were thoroughly mixed and aliquoted into PCR tubes (100 μL each) that were then heated at the designated temperature for 3 min (44 °C to 56 °C), cooled at room temperature for 3 min, and finally placed at −70 °C to quickly freeze. The cells were freeze-thawed three times in liquid nitrogen. The resulting cell lysates were centrifuged to isolate the soluble protein from cell debris. The resulting clear supernatant was mixed with a corresponding volume of loading buffer, and the mixture was subjected to immunoblotting as in a previous report [52].

### 4.10. Statistical Analysis

Each bar graph in this paper represents the mean ± standard deviation (SD) of triplicate individual experiments. For statistical analysis, all experimental data were analyzed by Mann–Whitney or ANOVA with a post hoc test followed by a Student–Newman–Kreuls test. *p*-values < 0.05 and < 0.01 were considered statistically significant. All statistical tests were performed with SPSS software (SPSS Inc., Chicago, IL, USA).

## Figures and Tables

**Table 1:** Primers sequences for the analysis of mRNA prepared from RAW264.7 cells used in RT-PCR.

Name	Direction	Sequence (5′ to 3′)
IL-2	Forward	GTGCTCCTTGTCACCAGCGC
	Reverse	GAGCCTTATGTGTTGTAAGC
MMP-9	Forward	TCTTCCCCAAAGACCTGAAA
	Reverse	TGATGTTATGATGGTCCCAC
COX-2	Forward	GGGAGTCTGGAACATTGTGAA
	Reverse	GCACATTGTAAGTAGGTGGACTGT
MCP-1	Forward	ACTGAAGCCAGCTCTCTCTT
	Reverse	ACGGGTCAACTTCACATTCA
ICAM1	Forward	CAGATGCCGACCCAGGAGAG
	Reverse	ACAGACTTCACCACCCCGAT
GAPDH	Forward	CAATGAATACGGCTACAGCAAC
	Reverse	AGGGAGATGCTCAGTGTTGG
**Primers Sequences Used in Real-Time PCR**		
COX-2	Forward	CCAGCACTTCACGCATCAGT
	Reverse	ACGCTGTCTAGCCAGAGTTTCAC
TNF-α	Forward	TGCCTATGTCTCAGCCTCTT
	Reverse	GAGGCCATTTGGGAACTTCT
IL-6	Forward	GCTGGAGTCACAGAAGGAGTGGC
	Reverse	GGCATAACGCACTAGGTTTGCGC
GAPDH	Forward	AGGGAGATGCTCAGTGTTGG
	Reverse	CAATGAATACGGCTACAGCA

## Data Availability

The data used to support the findings of this study are available from the corresponding author upon request.

## Sample Availability

Sample of the compound, Oe-ME, is available from the authors.
